# How Does Mentoring Affect the Creative Performance of Mentors: The Role of Personal Learning and Career Stage

**DOI:** 10.3389/fpsyg.2021.741139

**Published:** 2021-10-14

**Authors:** Shuang Xu, Pingqing Liu, Zheng Yang, Zunkang Cui, Fang Yang

**Affiliations:** School of Management and Economics, Beijing Institute of Technology, Beijing, China

**Keywords:** career stage, relational job learning, creative performance, mentoring, personal learning, personal skill development

## Abstract

Mentoring has become a vital strategy for improving employee performance and organizational development. A few previous literature studies made a detailed study on the benefits of mentees. The creative performance of mentors that improves from mentoring, however, only draws little attention. This article extends this line of inquiry by shedding light on whether, how, and when mentoring affects the creative performance of mentors, which is a crucial topic in research and practice. Based on the conservation of the resources theory (COR) and relational cultural theory (RCT), this article investigates the influence mechanism and boundary conditions of mentoring on the creative performance of mentors by conducting a multisource empirical study in China. The result shows that there is a positive impact of mentoring on the creative performance of mentors. We also reveal that the relationship is mediated by personal learning, especially the relational job learning dimension. Furthermore, this article finds that the career stage of mentors moderates the relationship between mentoring and personal learning, namely, the relationship is stronger among mentors at the later career stage. The empirical findings show implications for an understanding of how the creative behavior of mentors benefits mentoring and can be beneficial for developing the targeted measures to promote competitive advantages for organizations.

## Introduction

For the past several decades, mentoring has received increasing attention from management scholars and practitioners (Yi et al., 2017; Hu et al., 2020). Previous studies have revealed the positive effect of mentoring on the outcomes of mentees (Lapointe and Vandenberghe, 2017; Chen et al., 2020; Zheng et al., 2020). Beyond the benefits of mentees, mentors may also broaden their knowledge and get inspiration when they contribute energy and time to help mentees (Ragins et al., 2017; Ghosh et al., 2020). Several researchers have shifted their focus on the outcome variables of mentors that result from participating in mentoring, for example, job performance (Fowler et al., 2021), leadership capacity (Chun et al., 2012), and career development (McNall et al., 2010; Ghosh and Reio, 2013). However, they were largely restricted to the in-role performance of mentors. Mentors may acquire the resources that contribute to their creative performance from mentoring while the existing literature about this question is still less clear. It is still not known whether mentoring affects the extra-role performance of mentors, including the creative performance that has been regarded as a crucial predicator of organizational development. This oversight needs to be addressed because mentors are easily viewed as an agent of organization and the role modeling of mentees (Newman et al., 2018), and their creative performance is a key driver of organizational innovation. Thus, this study aims to address this gap by exploring the relationship and influence mechanism between mentoring and the creative performance of mentors.

In the mentoring literature, personal learning is regarded as a key resource that mentors benefit from mentoring, such as enhancing an understanding of the relationships and skills in organizations (Liu et al., 2009). Through guidance giving and constant contact with mentees, mentors can get skills to refresh knowledge, optimize management styles, and show insights into how they were perceived by others (Baluku et al., 2020), that is, enhancing personal learning. Then, this personal learning can promote the creative performance of mentors because it enables mentors to recognize a broader set of solution possibilities and generate novel and useful ideas. Mentors with effective personal learning can also better understand how to make full use of relationships to promote new ideas. Evidence also supports that personal learning is an important mediator for shedding light on the impact of mentoring on the performance of mentors (Powell and Greenhaus, 2010; Burmeister et al., 2020; Hu Y. et al., 2021). Therefore, we adopt personal learning (i.e., relational job learning and personal skill development) as a mediator in the relationship between mentoring and the creative performance of mentors.

Furthermore, the career stage of mentors may play an important role in the mediation mechanism. According to the conservation of resources theory (COR; Hobfoll et al., 2018), the resources level and value differ across the career stages of an individual (Kooij and Van De Voorde, 2011; Salmela-Aro and Upadyaya, 2018). Individuals tend to attach more importance to high-quality social relationships in their later career stage while individuals at the earlier career stage expect more about capability development, leading the skills to be more salient (Goštautaite et al., 2020). This may lead to a discrepancy between mentors in earlier and later career stages, especially regarding the learning resources. The relationship between mentoring and relational job learning tends to be stronger for later career mentors, while that with personal skill development may be stronger for earlier career mentors. Therefore, we examine a moderating role of career stage plays in the relationships between mentoring and personal learning, testing a moderating role of the career stage in the mediation effect of personal learning.

In this research, we aim to answer the question of whether, when, and how mentoring affects the creative performance of mentors. Although some qualitative studies proposed that one's creativity may increase while mentoring others (Newman et al., 2018), quite a few research studies paid attention to the impact of mentoring on mentors themselves. We examined the influence and mechanism of mentoring provided on the creative performance of mentors based on the COR and relational cultural theory (RCT; Fletcher and Ragins, 2007). In line with previous studies suggesting that a mentor is a person who has a stronger resource pool in the organization and conforms to organization values (Haggard et al., 2010), we argue that mentoring can be instrumental for mentors in acquiring additional valued resources, which can be utilized in creative behaviors.

**Figure 1:**
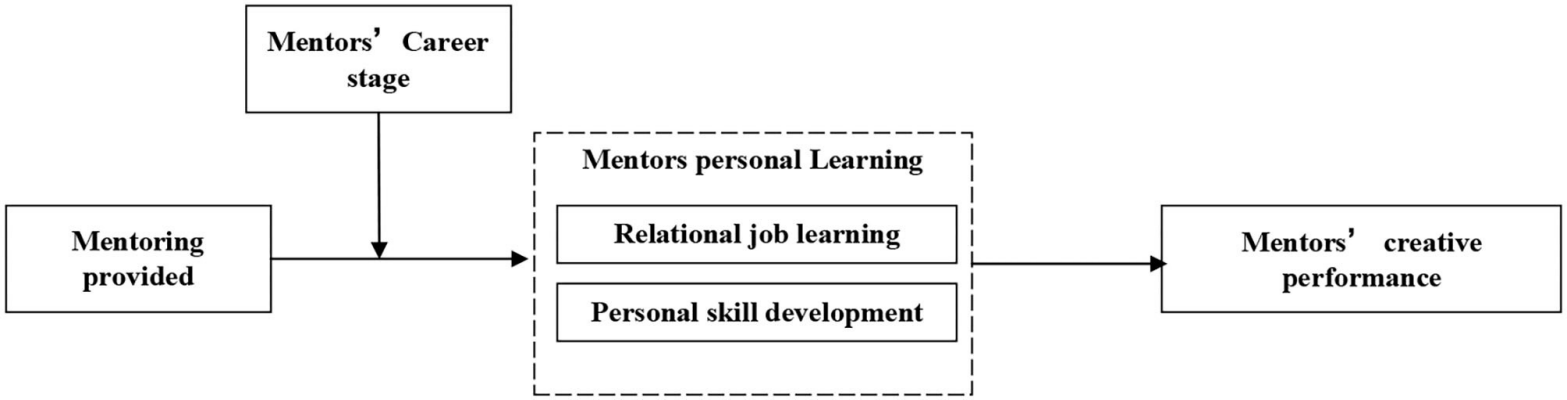
A conceptual framework.

This article contributes to the previous literature in three aspects. First, as far as we know, this is the first empirical study to examine the influence of mentoring on the creative performance of mentors, which broadens the benefits of mentors from mentoring into extra-role performance. Second, this study explores a mediating role of personal learning, including relational job learning and personal skill development, which is conducive to investigate mentoring and the creative performance of an employee from the perspective of resources. Finally, it is one of the studies that examine a moderating effect of the career stage of mentors, which reveals that mentors at the earlier career stage were more committed to career development while those at the later career stage were motivated by involvement. This encourages future researchers to consider the career stage in the application of the COR theory in the mentoring literature. The conceptual model is presented in Figure 1.

## Hypotheses Development

### Mentoring and the Creative Performance of Mentors

Employee creative performance refers to the development of novel and useful ideas about products, practices, services, or procedures (Zhang et al., 2020). The amount of mentoring provided by mentors has positive effects on their creative performance for three reasons. First, providing mentoring can stimulate mentors to generate more new and useful ideas in the workplace. The more mentoring that mentors provide in the organization, the more and various connections they can build with others from different backgrounds. This helps mentors to improve their cognitive process, gain growth in prosocial traits such as problem-solving skills, and be more flexible in thinking (Lee et al., 2010; Wasburn-Moses et al., 2014). In addition, as mentors need to answer the questions of mentees and “teaching by doing,” mentors can be easier to acquire the updated information and get access to the problems in the existing plans. These cognition and motivation improvements are beneficial for generating useful solutions in the workplace.

Second, providing mentoring can enhance the abilities of mentors in promoting ideas. Mentors are described as influential individuals with advanced work experience in the workplace (Scandura and Williams, 2001; Fowler et al., 2021). Having strong ties with others can amplify their influence, contributing to the promotion and implementation of ideas (Yang T. et al., 2021). A few literature studies have claimed that the Chinese mentor-mentee relationship possesses strong ties by stressing intimacy, reciprocity, and hierarchy (Zhou et al., 2019). Thus, mentors can have more opportunities to utilize this relationship to promote and practice ideas, which are beneficial for creative performance.

Finally, previous empirical research has also shown that employees can enhance creative performance from the complex social interaction among the actors in the social network (Perry-Smith and Mannucci, 2015; Zhou and Wu, 2018), for example, the mentoring relationship that has the attribute of a consistent interaction (Sun et al., 2014; Yang F. et al., 2021). Thus, the amount of mentoring provided by mentors can enhance the creative performance of mentors. Following these arguments, we put forward Hypothesis 1.

*H1*. The amount of mentoring provided by mentors positively relates to creative performance.

### A Mediating Effect of Personal Learning

Research has shown that sufficient resources are necessary for employees to improve creative performance, in which learning is one of the key resources (Ghosh and Reio, 2013). According to the mentoring literature and RCT, both mentors and mentees may experience mutual learning in mentoring (Hu Z. et al., 2021). Researchers also found that interacting with others influences personal learning and then affects employee behavior (Hu Y. et al., 2021). Therefore, we hypothesize that personal learning may mediate the relationship between providing mentoring and the creative performance of mentors. Personal learning is defined as an increase in the understanding of knowledge acquisition, skills, or competencies contributing to individual development and growth, including relational job learning and personal skill development (Liu and Fu, 2011; Jiang et al., 2016). Relational job learning focuses on the relationship aspect, referring to the increased understanding of the working environment and an interdependent relationship with others in the organization (Kwan et al., 2010). Personal skill development emphasizes the skills perspective and refers to the acquisition of new skills or abilities that can promote working relationships, such as communicating effectively and listening attentively (Weinberg, 2019; Hu Y. et al., 2021).

Previous studies have noted that personal learning is a proximal outcome that mentors gain from mentoring (Hu Y. et al., 2021). Personal learning can explain why mentors gain growth from mentoring others and show how the learning resources influence individual behavior. RCT emphasizes that every relationship or connection with others (for example, mentor-mentee relationship) can regenerate more resources for individual growth and development (Guy and Arthur, 2020). Mentors are more easily to make progress when they involve in intimate connections with mentees. In the context of the collective culture of China, the Chinese mentor-mentee relationship is a binary state with a mutual growth in connection. Mentoring presents opportunities for mentors to observe organizations from the perspective of mentees (Liu et al., 2009), strengthening their frequency and the quality of social communication. This allows the mentors to better understand interpersonal interaction in the workplace. Through self-reflection, mentors gain relational job learning. In addition, as mentoring contains lots of information exchange between mentors and mentees, the abilities of mentors to interact with others and solve problems can be improved. This will promote the personal skill development of mentors. In summary, the amount of mentoring provided by mentors will be positively related to their learning.

Furthermore, the personal learning of mentors can enhance their creative performance. According to the COR theory, sufficient resources can encourage employees to conduct behaviors that may not be formally required and be more willing to participate in a challenging task. The expansion of personal resources may influence the creative performance of mentors by increasing their ability and willingness to be creative in the workplace. First, mentors having increased personal skill development can be more exposed to update knowledge and problem diagnosis (Hobfoll et al., 2018). This continuous acquiring or reactivating of a store of knowledge is quite helpful for mentors to propose more new and useful ideas. Second, the relational job learning of mentors can help them to identify potential allies such as sponsors and establish a coalition of advocates. As mentors with increased relational job learning tend to hold positive views on their roles in the organizations, they can be more willing to step out of mental frames. This relational resource leads mentors to activate their creative potential by exerting themselves in promoting creative ideas. Thus, we suggest that mentors with personal learning resources are more likely to allocate and utilize resources to engage in creative tasks, which are positively related to their creative performance.

Given the abovementioned arguments, we suggest that the personal learning of mentors is positively related to their creative performance and that the level of personal learning is often enhanced by providing mentoring. By integrating the COR theory and RCT, we claim an integrated framework and argue that mentors are easily increasing resources on self-learning when providing mentoring. Mentoring others give mentors more opportunities to be exposed to others and contact with updated insights (Fowler et al., 2021). More resources encourage mentors to be more creative in the workplace. The personal learning of mentors can further active their creative potential as they can have increasing knowledge, ability, and a willingness to generate and promote new ideas, which can eventually improve the creative performance of mentors. Therefore, we propose that personal learning plays a mediating role between mentoring and the creative performance of mentors, and put forward Hypothesis 2.

*H2*. (a) Relational job learning and (b) personal skill development mediate the relationship between the amount of mentoring provided by mentors and their creative performance.

### A Moderating Role of the Career Stage of Mentors

Furthermore, according to the COR theory, as individuals at different career stages experience different resource levels and needs/goals, we hypothesize that the role of personal learning can be moderated by the career stage of mentors (Demerouti et al., 2012). The career stage literature has suggested that employees at the later career stage tend to report a higher level of available resources and have stronger prosocial motives than those at the earlier career stage (Kooij and Boon, 2018). Relational job learning emphasizes an understanding of interdependent of one's job related with others, which is more fit to the needs of mentors at the later career stage. Mentoring enables them to expand social circles and get more macroscopic information related to work, producing an overall view of interpersonal relationships. These benefits can be more prominent for mentors at the later career stage as they tend to develop a more efficient cope mechanism (Chen et al., 2012) while those at the earlier career stage put more emphasis on career development and involvement (Hess and Jepsen, 2009; Goštautaite et al., 2020). Therefore, the career stage of mentors plays a moderating role in the relationship between the amount of mentoring provided by mentors and their relational job learning, and the effect is stronger for mentors at the later career stage. Similarly, the increment of personal skill development can also be moderated by the career stage of mentors. Mentors at the earlier career stage mainly aim to build up expertise in the organization. Mentoring gives them more opportunities to practice knowledge and skills, which is conducive for their communication and problem-solving abilities. In other words, the career stage of mentors can moderate the impact of the amount of mentoring on the personal skill development of mentors, and the effect is stronger for mentors at the earlier career stage. In line with these arguments, we put forward Hypothesis 3.

*H3*. Mentor's career stage moderates the amount of mentoring provided by mentors—personal learning relation.
*H3a*. Mentor's career stage moderates the relationship between the amount of mentoring provided by mentors and relational job learning, such that this relationship is stronger (vs. weaker) for later-career mentors (vs. earlier-career mentors).
*H3b*. Mentor's career stage moderates the relationship between the amount of mentoring provided by mentors and personal skill development, such that this relationship is stronger (vs. weaker) for earlier-career mentors (vs. later-career mentors).

We have so far demonstrated a mediating role of personal learning (H2) and a moderating role of career stage (H3) in the relationship between the amount of mentoring provided by mentors and their creative performance. These combined effects indicate the presence of a moderated mediation model, whereby the career stage moderates the indirect effect of personal learning. Thus, we postulate that the amount of mentoring provided by mentors may have a stronger influence on the relational job learning for mentors at later career stages while the role of the amount of mentoring provided by mentors on personal skill development may proportionally more important for mentors at earlier career stages. Given this, we put forward Hypothesis 4.

*H4*. Mentor's career stage moderates the mediation effect of personal learning.
*H4a*. Mentor's career stage moderates the mediation effect of relational job learning, such that the effect is stronger (vs. weaker) for later-career mentors (vs. earlier-career mentors).
*H4b*. Mentor's career stage moderates the mediation effect of personal skill development, such that the effect is stronger (vs. weaker) for earlier-career mentors (vs. later-career mentors).

## Methods

### Participants and Procedures

To examine the proposed hypotheses, we collected the questionnaire data from 15 enterprises located in the different provinces of China, which have a long space distance. These enterprises asked the more experienced employees to provide guidance and support to less knowledgeable individuals. The experienced employees are in line with the established definition of the mentor (Ragins et al., 2000). These data provide detailed information on mentoring. To ensure the authenticity and accuracy of the questionnaire, we communicated with HR departments of those companies in advance and obtained a list of mentors. Surveys were distributed to randomly selected mentors and their supervisors to eliminate the concerns of respondents, including an explanation of the purpose of this study and assurances regarding anonymity and how the data would be used. To avoid a common method bias, we divided the questionnaire into types A and B: A was the report of mentors on their mentoring provided, personal learning, and demographic variables; B was the evaluation of the direct supervisors of mentors on the creative performance of their corresponding subordinates. To match the data of mentors with their supervisors, we have coded paired questionnaires of mentors and their supervisors at the upper left corner of questionnaires before distribution. Mentors provided their code to their supervisor who filled it when completing his/her questionnaire.

About 200 questionnaire packages—consisting of a mentor questionnaire and a supervisor questionnaire—were distributed to mentors and their supervisors. Of the 165 packages that were returned, 153 were usable (response rate 92.727%). The response rate was satisfactory, considering that the missing data seemed to be non-systematic and were well below missing-at-random rates (e.g., 40%; Raymond and Roberts, 1987). Of the mentors, 83.7% were men and 16.3% were women. The average age was 32.15 years (SD = 6.39 years, range = 24–54), and organizational tenure was 8.82 years (SD = 6.71 years). The sample was considerably educated as 8.1% of the respondents held postsecondary school qualifications, 68.6% held undergraduate degrees, and 23.3% held graduate degrees. Because the variables included in the study are mostly well-documented constructs in the extant literature, we adopted the established measures that acknowledge psychometric properties. A 6-point response format (1: not at all, 6: to a great extent) was used for all substantive items.

### Measures

#### Mentoring Provided

Mentoring provided refers to the amount of mentoring that an employee has provided over their career history within an organization (Bozionelos, 2004). We measure the degree of mentoring provided by the mentor using Bozionelos's (2004) 6-item scale (sample items are “whom I have consistently provided emotional support” and “I was personally interested in his or her professional development”). Cronbach's alpha reliability coefficient was 0.914.

#### Creative Performance

The creative performance of mentors was measured by a 4-item scale put forward by Farmer et al. (2003). An example item was “he will seek new ideas/ways to solve problems.” Cronbach's alpha reliability coefficient was 0.936.

#### Personal Learning

We used a scale developed by Lankau and Scandura (2002), assessing the two dimensions of personal learning—namely, relational job learning and personal skill development. Each dimension has six items. A sample item to measure relational job learning was “I have increased my knowledge about the organization as a whole in the workplace” while a sample item to measure personal skill development was “I have become more sensitive to others' feelings and attitudes in the workplace.” Cronbach's alphas for relational job learning and personal skill development were 0.868 and 0.875, respectively.

#### Career Stage

The career stage has been measured by multiple indictors, such as chronological age, professional tenure, or organizational tenure (Kooij and Van De Voorde, 2011). In this article, we asked mentors to indicate their organizational tenure in years and used it as a proxy for the career stage. Considering the literature and sample conditions, the career stage was defined by three categories: earlier career stage (<2 years), middle career stage (between 2 and 10 years), and later career stage (more than 10 years). These categories are in line with those used in other similar researches on organizational psychology (Kooij and Boon, 2018). The career stage was coded on a 3-point scale where the higher scores indicate later stages.

#### Control Variables

Because the degree to which the creative performance of employees might be related to the demographic variables (Becker, 2005), we controlled these variables, including the mentors' age, gender (dummy coded: 0 = female, 1 = male), organizational tenure (years), and education level (coded on a four-point scale: from 1 = junior college or lower to 4 = doctoral degree or above).

### Analytical Approach

To test the hypotheses, we first adopted a confirmatory factor analysis (CFA) to test the distinctiveness of variables. MPLUS 7.4 was used, and the indices, such as root mean square error of approximation (RMSEA), comparative fit index (CFI), Tucker–Lewis index (TLI), standardized root mean square residual (SRMR), were chosen to represent the goodness-of-fit. Second, we conducted a multiple regression test to find the impact of mentoring provided on the creative performance of mentors (H1). Thirdly, we tested a mediating effect of personal learning (i.e., relational job learning and personal skill development) using hierarchical regression analysis and the bootstrapping methodology (H2). Bootstrap can do repeated samples from the research samples, which can make the parameter estimation of the model more accurate, robust, and reliable. We obtained the 95% bootstrapped CI (5,000 bootstrap samples) with the aid of the statistical package for the social sciences (SPSS) PROCESS Macro Model 4. Finally, we examined the moderation effect of career stage on personal learning using hierarchical regression analysis (H3) and further tested the moderated mediation hypothesis with an aid of the PROCESS Macro with 5,000 bootstrap samples (H4). PROCESS is a syntax developed by Hayes (2013) to analyze mediation and moderation models as well as their combination. This approach more accurately reflects mediation and moderated mediation effects (Hayes, 2018). For further analysis of the interaction effects, we calculated simple slopes for three career stages, earlier career (one SD below the mean organizational tenure: 2 years), middle career (mean organizational tenure: 9 years), and later career (one SD above the mean organizational tenure: 16 years). All variables were mean-centered before the analysis.

## Results

Table 1 shows the descriptive statistics and correlations for the study variables and control variables. It can be seen that mentoring provided was significantly positively correlated with the creative performance of mentors (*r* = 0.550, *p* < 0.001), relational job learning (*r* = 0.368, *p* < 0.001), but not with personal skills development (*r* = 0.146, *p* > 0.05). Similarly, the creative performance of mentors was significantly positively correlated with relational job learning (*r* = 0.318, *p* < 0.001) but not with personal skill development (*r* = 0.075, *p* > 0.05).

**Table 1:** Means, SDs, and correlations.

**Variable**		**Mean**	**SD**	**1**	**2**	**3**	**4**	**5**	**6**	**7**	**8**
1	Mentoring provided	4.729	1.054	1.000	0.550^***^	0.368^***^	0.146	0.115	−0.174^*^	0.018	0.074
2	Creative performance	4.769	0.893		1.000	0.318^***^	0.075	0.094	−0.185^*^	0.055	−0.119
3	Relational job learning	4.594	0.788			1.000	0.386^***^	0.013	−0.154	−0.053	−0.013
4	Personal skill development	4.835	0.991				1.000	−0.008	−0.213^**^	−0.130	−0.107
5	Gender	0.837	0.371					1.000	−0.075	0.308^***^	−0.113
6	Age	32.150	6.390						1.000	−0.141	0.220^***^
7	Education	2.151	0.543							1.000	−0.371^***^
8	Organizational tenure	8.820	6.710								1.000

* *p < 0.05*,

** *p < 0.01*,

*** *p < 0.001*.

To check the discriminant validity of the four latent variables (mentoring provided, relational job learning, personal skill development, and creative performance), we conducted a series of CFAs. As shown in Table 2, the baseline four-factor model (Model 1 in Table 2) fits the data better compared to other alternative models (χ^2^/*df* = 1.903; RMSEA = 0.078; CFI = 0.921; TLI = 0.903; SRMR = 0.069). In Models 2–7, items for any two variables loaded on a common factor, and others loaded on their factor. In Models 8–11, items for any three variables loaded on a common factor, and others loaded on their factor. In Model 12, all items are loaded on one single factor. The CFA results supported the discriminant validity of the key variables.

**Table 2:** A summary of model fit indices.

**Model**	**χ^2^/df**	**CFI**	**TLI**	**RMSEA**	**SRMR**
4-Factor: MP; CP; JL; SD	1.903	0.921	0.903	0.078	0.069
3-Factor: MP + JL; CP; SD	3.389	0.787	0.743	0.127	0.107
3-Factor: MP + CP; JL; SD	4.164	0.718	0.659	0.147	0.210
3-Factor: MP + SD; JL; CP	2.804	0.839	0.806	0.111	0.092
3-Factor: CP + JL; MP; SD	3.993	0.734	0.678	0.143	0.233
3-Factor: CP + SD; MP; CP	4.112	0.723	0.665	0.145	0.241
3-Factor: JL + SD; MP; CP	2.425	0.873	0.847	0.098	0.080
2-Factor: MP + CP + JL; SD	6.071	0.544	0.454	0.186	0.291
2-Factor: MP + JL + SD; CP	6.716	0.435	0.385	0.197	0.306
2-Factor: CP + JL + SD; MP	5.432	0.601	0.523	0.174	0.280
2-Factor: SD + MP + CP; JL	6.118	0.540	0.449	0.187	0.283
Singer-factor	7.257	0.434	0.326	0.206	0.305

*MP, mentoring provided; JL, relational job learning; SD, personal skill development; CP, creative performance*.

### The Relationship Between Mentoring Provided and Creative Performance of a Mentor

To test the main effect of mentoring provided on the creative performance of a mentor, this article tested *H1 via* hierarchical regression. To minimize the threat of multicollinearity, all variables were z-standardized before entering them into the regression equations. The evaluations of the regression assumptions of normality and linearity, as well as the absence of multicollinearity, were satisfactory. *H1* asserted that mentoring provided by mentors has a positive impact on the creative performance of mentors. Thus, we tested the influence of mentoring provided on mentor creative performance. Table 3 shows the relationship between mentoring provided and the creative performance of a mentor under the control of gender, age, education, and occupational experiences. The results suggest that mentoring provided had a positive impact on the creative performance of a mentor (β = 0.537, *p* < 0.001).

**Table 3:** Hierarchical regression results.

**Variable**	**Mentors' creative performance**	
	**Model 1**	**Model 2**
Gender	0.072	0.044
Age	−0.143	−0.089
Education	0.022	0.033
Organizational tenure	−0.087	−0.030
Mentoring provided		0.537^***^
R-sq	0.050	0.329
Adjusted R-sq	0.021	0.303
ΔR-sq	0.050	0.279
F	1.696	12.567^***^

*** *p < 0.001*.

### The Mediation Effect of Personal Learning

To examine the mediation effect of the specific dimensions of personal learning, we test *H2a* and *H2b*. First, we tested the mediation effect of relational job learning. In the first step of mediation regression, we regressed relational job learning on mentoring provided. As shown in the second row of Table 4 (M4), the amount of mentoring provided by mentors can positively affect their relational job learning (β = 0.322, *p* < 0.001). In the second step, we regressed the creative performance of a mentor on the relational job learning, and the fifth row of Table 4 (M7) shows that relational job learning can positively influence the creative performance of a mentor (β = 0.296, *p* < 0.001). In the final step, we regressed the creative performance of a mentor on mentoring provided and relational job learning. As shown in the seventh row of Table 4 (M9), mentoring provided by a mentor and mentor relational job learning can positively impact their creative performance at the same time (β = 0.499, *p* < 0.001; β = 0.147, *p* < 0.05). Thus, relational job learning plays a mediating role in the influence of mentoring provided and the creative performance of a mentor, which indicates that *H2a* is supported.

**Table 4:** Mediation regression results.

**Variable**	**Relational job learning**		**Personal skill development**		**Mentors' creative performance**			
	**Model 3**	**Model 4**	**Model 5**	**Model 6**	**Model 7**	**Model 8**	**Model 9**	**Model 10**
Gender	0.002	−0.014	0.029	0.027	0.052	0.054	0.027	0.032
Age	−0.060	−0.028	−0.137	−0.133	−0.104	−0.121	−0.116	−0.076
Education	−0.050	−0.047	−0.248^**^	−0.247^**^	0.046	0.030	0.010	0.032
Organizational tenure	−0.188	−0.145	−0.270^**^	−0.264^**^	−0.071	−0.120	0.002	−0.066
Mentoring provided		0.322^***^		0.045			0.499^***^	0.534^***^
Relational job learning					0.296^***^		0.147^*^	
Skill development						0.002		−0.022
R-sq	0.045	0.145	0.136	0.138	0.136	0.052	0.344	0.329
Adjusted R-sq	0.016	0.111	0.109	0.104	0.102	0.015	0.315	0.296
ΔR-sq	0.045	0.099	0.136	0.002	0.050	0.002	0.208	0.277
F	1.528	4.332^***^	5.062^***^	4.084^**^	3.982^**^	1.389	11.647^***^	10.174^***^

* *p < 0.05*,

** *p < 0.01*,

*** *p < 0.001*.

**Table 5:** Direct and indirect effects.

**Path**	**Effect**	**95% CI**	
		**LLCI**	**ULCI**
Direct effect: Mentoring provided → Creative performance	0.436	0.308	0.563
Indirect effect:			
Mentoring provided → Relational job learning → Creative performance	0.049	0.001	0.111
Mentoring provided → Personal Skill development → Creative performance	−0.003	−0.021	0.011

Second, we tested the mediating effect of personal skill development. In the first step, we regressed personal skill development on mentoring provided. As is shown in the fourth row of Table 4 (M6), the effect of mentoring provided on their personal skill development was not significant (β = 0.045, *p* > 0.05). In the second step, we regressed the creative performance of a mentor on personal skill development and the sixth row of Table 4 (M8) shows that the impact of personal skill development on the creative performance of a mentor was not significant (β = 0.002, *p* > 0.05). In the final step, we regressed the creative performance of a mentor on mentoring provided and personal skill development. As shown in the eighth row of Table 4 (M10), the impact of personal skill development on the creative performance of a mentor was not significant (β = −0.022, *p* > 0.05) while the influence of mentoring provided was significant (β = 0.0534, *p* < 0.001). Thus, the mediation role of personal skill development was not significant.

Finally, to reveal the relationships among various mediating variables in the hypothesized model, we tested the mediation effect of the specific dimensions of personal learning simultaneously. The significance of this indirect effect was tested using bootstrap analysis. About 5,000 bootstrapped resamples were created with a replacement from the original data to generate a 95% CI for the estimate of an effect (MacKinnon et al., 2002). An indirect effect of relational job learning was found to be significant (0.049, 95% CI = 0.001, 0.111). Thus, *H2a* was supported. Similarly, *H2b* asserted that the influence of mentoring provided on the creative performance of a mentor would be mediated by personal skill development. Using 95% CIs for the estimates of indirect effects, this effect was not found to be significant (−0.003, 95% CI = −0.021, 0.011), not supporting *H2b*. Thus, *H2* was partly supported.

### The Moderation Effect of Career Stage of Mentors

**Figure 2:**
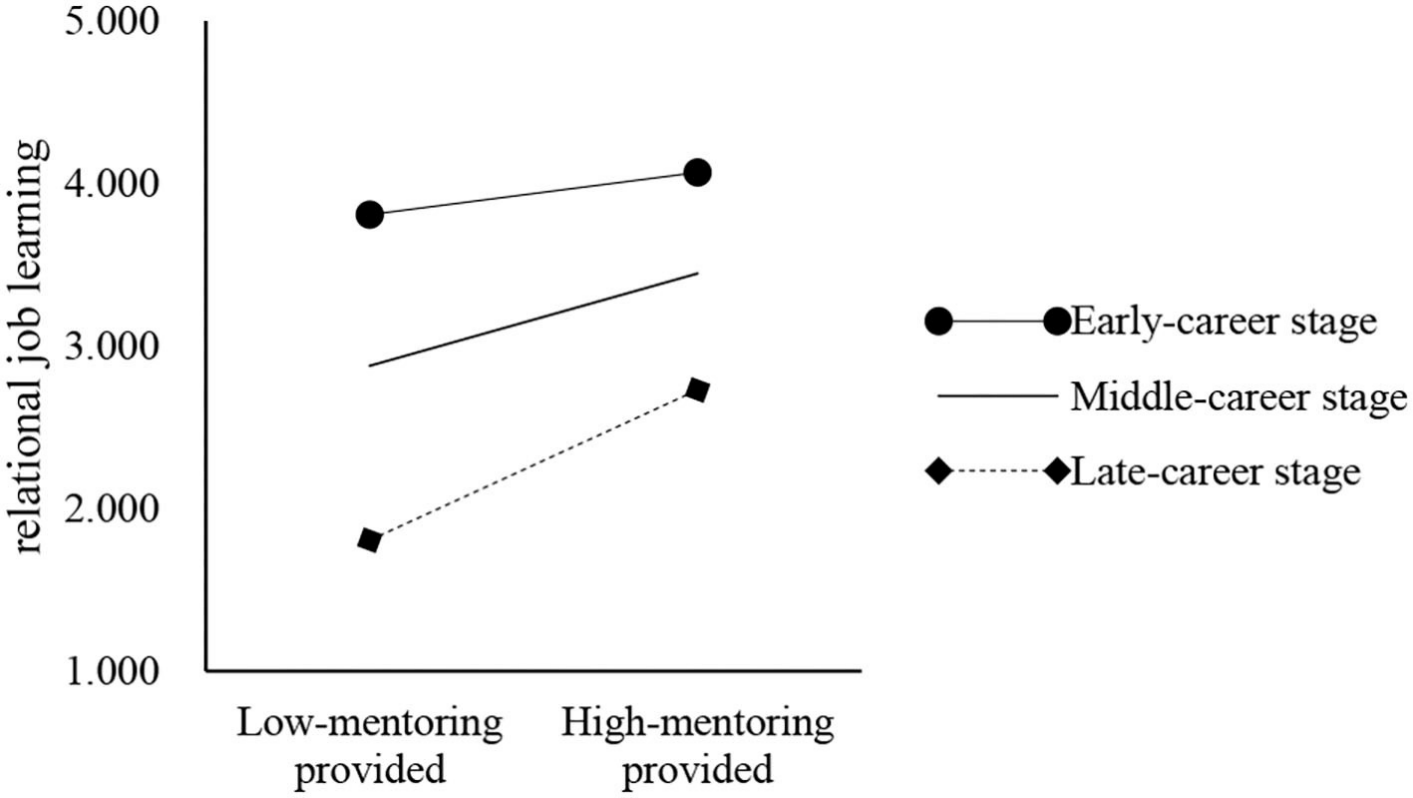
The moderation effect of career stage on the relationship between mentoring provided and relational job learning.

To directly test the proposed moderation model, a regression-based path analysis was used with the aid of existing computational tools for estimating and probing conditional effects in moderation models. As shown in Table 6, when the dependent variable is relational job learning, the interaction term of mentoring provided and career stage is significant (β = 0.163, *p* < 0.001), and when the dependent variable is personal skill development, the interaction term is not significant (β = −0.079, *p* > 0.05). Thus, the career stage of mentors can moderate the relationship between mentoring provided and relational job learning. The conditional effect is graphically depicted in Figure 2. It shows that the slope for mentors at the later career stage was steeper than the simple slope for those at the earlier career stage. Together, *H3* was partially supported.

**Table 6:** Moderation effect of the career stage of mentors.

**Variable**	**Relational job learning**	**Personal skill development**
Mentoring provided	0.290^***^	0.097
Mentor career stage	−0.819^***^	−0.122
Mentoring provided × career stage	0.163^**^	−0.006
R-sq	0.157	0.053
F	5.077^***^	1.510

** *p < 0.01*,

*** *p < 0.001*.

To explore a moderated mediation effect, we then used the PROCESS bootstrap method (Hayes, 2018). As shown in Table 7, the mediation effect of relational job learning for the earlier career stage was not significant (0.025, 95% CI = −0.006, 0.074), whereas the mediation effect for the middle and late career stage group was both statistically significant (0.055, 95% CI = 0.007, 0.119; 0.090, 95% CI = 0.010, 0.194). In addition, the relationship for the middle career stage group was not as strong as for the late career stage group. Thus, when the mentor is at the later career stage, the mediation effect of relational job learning in the relationship between mentoring provided and creative performance comes to be stronger. Therefore, *H4* was supported.

**Table 7:** Results of a moderated mediation effect.

**Moderated mediation model**	**Effect**	**SE**	**95% CI**	
			**LLCI**	**ULCI**
Conditional indirect effects *via* relational job learning				
Earlier career stage	0.025	0.021	−0.006	0.074
Middle career stage	0.055	0.029	0.007	0.119
Later career stage	0.090	0.048	0.010	0.194
Conditional indirect effects via personal skill development				
Earlier career stage	−0.006	0.013	−0.042	0.010
Middle career stage	−0.006	0.012	−0.037	0.011
Later career stage	−0.006	0.014	−0.040	0.020
Index of moderated mediation				
Mediator				
Relational job learning	0.033	0.020	0.001	0.078
Personal skill development	−0.001	0.007	−0.012	0.016

## Discussion

An increasing number of studies discuss the benefits of mentors from mentoring (Mao et al., 2016; Ghosh et al., 2020), yet some research studies focusing on the creative performance of mentors and mentors at different career stages are still scarce. To address this gap, we developed a moderated mediation model to explore the relationship and underlying mechanisms between mentoring and the creative performance of mentors *via* personal learning. We also investigated the moderating role of the career stage in these relations. Our hypotheses were largely supported. The results provide evidence that mentoring positively relates to the creative performance of mentors, and relational job learning plays a mediation role in this relationship. Personal skill development, however, fails to act as a mediator. The results also showed a moderating role of the career stage of mentors in affecting the mediation effect of relational job learning of mentors. The hypotheses results are summarized in Table 8. The results of this study have both theoretical and practical implications.

**Table 8:** Results of hypotheses testing.

**Hypotheses**	**Coefficients**	**SE**	**T**	**P**	**95% CI**	**Remarks**
H1	0.537	0.065	7.300	0.000^***^	–	Supported (Table 3)
H2a	0.049	0.028	–	–	[0.001, 0.111]	Supported (Tables 4, 5)
H2b	−0.003	0.008	–	–	[−0.021,0.010]	Not supported (Tables 4, 5)
H3a	0.163	0.063	2.595	0.010^**^	–	Supported (Table 6, Figure 2)
H3b	−0.006	0.077	−0.075	0.940	–	Not supported (Table 6)
H4a	0.033	0.020	–	–	[0.001,0.078]	Supported (Table 7)
H4b	0.000	0.007	–	–	[−0.012,0.016]	Not supported (Table 7)

** *p < 0.01*,

*** *p < 0.001*.

### Theoretical Implications

The results of this article have three theoretical implications. First, we extend the mentoring theory into the creative performance of mentors. This study integrates the RCT and COR theory and reveals that mentoring in the workplace can stimulate the creative performance of a mentor through personal learning. Compared with previous studies that either focused on the benefits of mentees obtained from mentoring (Eby et al., 2013; Hu et al., 2020) or largely investigated the in-role performance of the mentors (Fowler et al., 2021), our finding broadens the application of the COR theory into the perspective of mentors to find that mentoring can lead mentors to gain resources as well. Theoretically, mentors can experience positive interactions, including mutual learning and collision of various ideas during mentoring provision (Lentz and Allen, 2009; Ghosh et al., 2020), which serve as valuable resources to promote their creative performance. This finding further supports previous studies of viewing reciprocity as one fundamental attribute in mentoring (Haggard et al., 2010). To our knowledge, this study is the first empirical research that links the amount of mentoring provided by mentors and their creative performance and reveals a positive relationship. The result can cover future research to address the impact of mentoring on the beneficial outcomes of mentors.

Second, this study supplements the mentoring literature by taking personal learning (and the specific aspects of it) as mediators in the relationships between mentoring and the creative performance of mentors, especially finding only relational job learning effectively. By exploring a mediating effect of relational job learning (relationship aspect) and personal skill development (skills aspect) simultaneously, we surprisingly find that only relational job learning is significant. Compared to these findings with previous studies, which took personal learning as one mediator in mentoring mechanism (Liu et al., 2009; Burmeister et al., 2020; Hu Y. et al., 2021), our results further indicate the differences in a mediating role that the two dimensions of personal learning plays in the relationship between mentoring and the creative performance of mentors. The explanation for an unexpected result may come from the different evaluation of resources in the Chinese context where there is a collectivist culture and high power distance. According to the COR theory, the value of resources depends on the degree that it can increase the fit between a person and his or her environment (Halbesleben et al., 2014). Chinese mentors tend to evaluate social ties and interaction as more valuable than Westerns. Although mentors can truly update knowledge and sharpen skills from providing mentoring, the value of skill development is less significant than that of relationship improvement. In addition, in the high-power distance culture, mentees incline to follow the lead of the senior employees, making the progress of Chinese mentors on skill development limited. Thus, this finding contributes to the mentoring literature and COR theory by revealing the significant value of relationship resources gaining for Chinese mentors in affecting their behavior and performance.

Thirdly, this study has highlighted the moderation role of the career stage of mentors and answered the call for taking the timing of resources in one's career into consideration (Halbesleben et al., 2014). Although previous research has found that the learning of mentors plays a mediation role in the relationship between mentoring and the outcomes of mentors (Lankau and Scandura, 2002), much less is known about the effect under different situations, such as career stage. Drawing on the COR theory and career-stage literature (Hall, 2002), we hypothesized that the mediation effect of personal learning of mentors may depend on the career stage. The results partially confirm the proposition as we find that the mediation effect of relational job learning (relationship aspect) was stronger at later career stages. Theoretically, the findings align with the argument that employees at the earlier career stage tend to be more committed to career development. Meanwhile, those at the later career stage were more committed by involvement. However, one unexpected finding was no significant difference in the mediation effect of skill development (skill aspect) among mentors at different career stages. This result may be explained by the fact that Chinese mentors and mentees tend to maintain a family-like relationship with the characteristics of intimacy and hierarchy (Zhou et al., 2019). It is much more than a working relationship, and mentees are more likely to respect and obey the suggestions of mentors, so providing mentoring has less skill development benefits for Chinese mentors. Mentees brought by the mentors at the earlier career stage are often more junior and have less capacity and desire to challenge mentors, which makes that despite the different skill resource values pursued by the mentors at different career stages, there is no significant difference regarding the skill aspect. Thus, we find the career stage of mentors mainly moderates a mediating role of this relationship aspect, and the effect will be stronger when mentors are at the later career stage. Our integration of COR with the career stage advances an understanding of the values of different resources from the career span perspective. This research can encourage future researchers to consider the issues of the career stage in the application of the COR theory in the mentoring literature.

### Practical Implications

This research has several practical implications for individuals and organizations. First, as mentoring has been regarded as a useful tool to promote competitive advantages for organizations, both mentors and organizations need to be aware of the effect of mentoring others in learning and creative performance. On one hand, organizations need to communicate the potential outcomes of mentoring and pay attention to the attitude and behavior of both mentors and mentees during mentoring implementation; on the other hand, mentors need to make full use of interaction with their mentees, activating the accumulated knowledge, which can enhance their creative performance and eventually facilitate their career development.

Second, the organization needs to realize the importance of relational job learning. The results showed a crucial role of relationships in promoting creative performance, thus, organizations need to bolster the perception of mentors of the expansion of social networks they own in the organization and give them more feedback on their mentoring effect. Simultaneously, the organization needs to help mentors set realistic relationship expectations, which may lead to better relationship outcomes.

Finally, this article claims that the career stage plays a moderating role in explaining the beneficial effect of mentoring, thus the career stage of mentors should be taken into consideration to achieve a high degree of effectiveness. The organization can pay more attention to improve the sense of the existence of mentors and meet their relationship needs, especially for those in a later career; while for those at the earlier career stage, the organization needs to raise their feeling on the recognition about their work and ability.

### Limitations and Future Directions

Although this article provides robust empirical evidence that mentors can gain creative performance from mentoring, it still possesses several limitations that enlighten future research. First, we explore the outcome of mentors by conducting cross-sectional research. It may lead to a limitation on the mediation test and reveals the stage difference of mentors. Future research can use the longitudinal data to further examine the effect of mentors' resource gaining from mentoring.

Second, although we extend mentoring research by integrating the COR theory and RCT to find the mediator of personal learning and the moderator of the career stage, there may be other theoretical perspectives lying in the influence mechanism. For example, future research can consider more unique variables in the Chinese context like supervisor-subordinate *guanxi* in China (Liu and Shi, 2017).

Finally, this article only uses a quantitative method to analyze the influence of mentoring. Although this method is suitable to verify the known theoretical hypotheses, future research can also adopt a qualitative method such as the case study to explore the mechanism of different elements of mentoring on the outcomes of mentors, which can help to further deepen mentoring research and the COR theory.

## Data Availability Statement

The raw data supporting the conclusions of this article will be made available by the authors, without undue reservation.

## Author Contributions

SX: conceptualization, software, formal analysis, and writing—original draft. PL: conceptualization, investigation, resources, writing—reviewing and editing, supervision, and project administration. ZY: methodology, investigation, data curation, and writing—reviewing and editing. ZC and FY: contributed to the interpretation of the results and investigation process. All authors discussed the results and contributed to the manuscript.

## Funding

This article was supported by National Office for Philosophy and Social Sciences of China (20BGL136).

## Conflict of Interest

The authors declare that the research was conducted in the absence of any commercial or financial relationships that could be construed as a potential conflict of interest.

## Publisher's Note

All claims expressed in this article are solely those of the authors and do not necessarily represent those of their affiliated organizations, or those of the publisher, the editors and the reviewers. Any product that may be evaluated in this article, or claim that may be made by its manufacturer, is not guaranteed or endorsed by the publisher.
